# Plant-Based Approaches for Inhibition of Advanced Glycation End Products Formation: Dietary Polyphenols

**DOI:** 10.3390/molecules31162928

**Published:** 2026-08-21

**Authors:** Seray Akalin-Saygili, Aylin Ayaz

**Affiliations:** Department of Nutrition and Dietetics, Faculty of Health Sciences, Hacettepe University, Ankara 06100, Turkey

**Keywords:** advanced glycation end products, dietary polyphenols, plant-based extract, glycation, Maillard reaction, functional foods

## Abstract

Advanced glycation end products arise during thermal processing and endogenous metabolism, contributing to oxidative stress, inflammation, and the progression of chronic diseases. Dietary polyphenols have emerged as promising advanced glycation end product (AGE) inhibitors through antioxidant activity, carbonyl trapping, metal chelation, and modulation of inflammatory pathways. This review summarizes current evidence on the effects of polyphenols in food systems and highlights how different polyphenol subclasses vary in their antiglycation potential depending on chemical structure, food matrix, and cooking conditions. Although experimental studies consistently demonstrate inhibitory effects, the translation to humans remains limited by low bioavailability, metabolic transformation, and heterogeneous analytical methods. Standardized AGE measurements are needed to improve mechanistic insight, and evaluations of dose–response relationships are needed to clarify their relevance in real-world diets. Future research should prioritize long-term human studies and practical culinary strategies to determine whether polyphenol-rich foods can meaningfully reduce dietary AGE exposure and support chronic disease prevention.

## 1. Introduction

Thermal processing is widely used in food production to enhance flavor, extend shelf life, and ensure safety [1]. One of the key reactions triggered during heating is the Maillard reaction. This non-enzymatic browning process contributes to desirable sensory properties by generating characteristic aromas and flavors. However, the same reaction also promotes the formation of potentially harmful compounds, particularly AGEs [2]. AGEs arise through the formation of chemical linkages between the carbonyl groups of reducing sugars and the free amino group within the structures of nucleic acids, proteins, or lipids [3,4]. In addition to their formation in foods, various types of AGEs are also synthesized endogenously in the human body [1,5,6]. The accumulation of AGEs within the body leads to the development of numerous diseases. Research findings demonstrate that the elevation of AGEs in the body leads to an increased state of oxidative stress, inflammation, protein aggregation and dysfunction in tissues, resulting in the indication of diabetes, cardiovascular disorders, and renal pathologies [1,4,7,8,9,10]. Reducing dietary AGE exposure and endogenous AGE formation may help prevent AGE accumulation and its associated adverse effects. However, the relative contribution of dietary and endogenous AGEs to the total body AGE burden remains a matter of ongoing debate. Although dietary AGEs have been associated with adverse metabolic outcomes, their physiological impact depends on multiple factors, including gastrointestinal absorption, metabolic transformation, renal clearance, and tissue distribution [11]. Current evidence suggests that endogenous AGE formation driven by hyperglycemia, oxidative stress, and inflammation may represent the predominant source of systemic AGEs under many pathological conditions [3,12,13]. Consequently, reducing dietary AGE intake should be considered a complementary strategy rather than a replacement for limiting endogenous AGE formation.

Polyphenols are a group of bioactive phytochemicals characterized by the presence of one or more phenol rings in their structure [14]. Polyphenols include a diverse category of compounds primarily derived from plant-based sources, including phenolic acids, flavonoids, stilbenes, and lignans. Current research suggests that polyphenols may reduce the formation of AGEs during food processing and suppress the endogenous synthesis of AGEs within the human body, particularly through their antioxidant and anti-inflammatory properties [15,16,17,18,19].

Previous reviews have largely focused either on the mechanisms of AGE formation or on the general antiglycation properties of natural compounds. However, limited attention has been given to integrating evidence from food model systems, the application of plant-based ingredients for reducing AGE formation during food processing, polyphenol subclass-specific mechanisms, bioavailability constraints, and clinical evidence within a single framework. Therefore, this review provides a comprehensive evaluation of dietary polyphenols as AGE inhibitors from food systems to human health outcomes while critically addressing their translational potential, current limitations, and future research priorities.

### Literature Search Strategy

This review was conducted to provide an up-to-date overview of the role of dietary polyphenols in inhibiting AGE formation. The literature search was primarily performed using the PubMed and Scopus databases. Publications available up to January 2026 were considered, with emphasis placed on recent evidence while also including seminal studies considered essential to the field.

The search included combinations of keywords such as advanced glycation end products, polyphenols, phenolic compounds, dietary AGEs, food, food processing, glycation inhibitors and plant extracts. Additional relevant studies were identified through the reference lists of key publications.

Priority was given to original research articles, experimental investigations, clinical studies, and high-quality review papers that addressed the formation of AGEs, the mechanisms of polyphenol-mediated inhibition, food applications, bioavailability, and health outcomes. Publications not directly related to dietary polyphenols or AGE inhibition, non-English articles, conference abstracts, editorials, and studies lacking sufficient methodological information were not considered.

## 2. Generation and Formation of Endogenous and Exogenous AGEs

Advanced glycation end products represent a heterogeneous group of compounds formed through several distinct chemical pathways. Based on their chemical properties, AGEs are categorized into three distinct groups. These are: non-fluorescent cross-linking compounds, fluorescent-crosslinking compounds, and non-crosslinking compounds [16].

**Figure 1:**
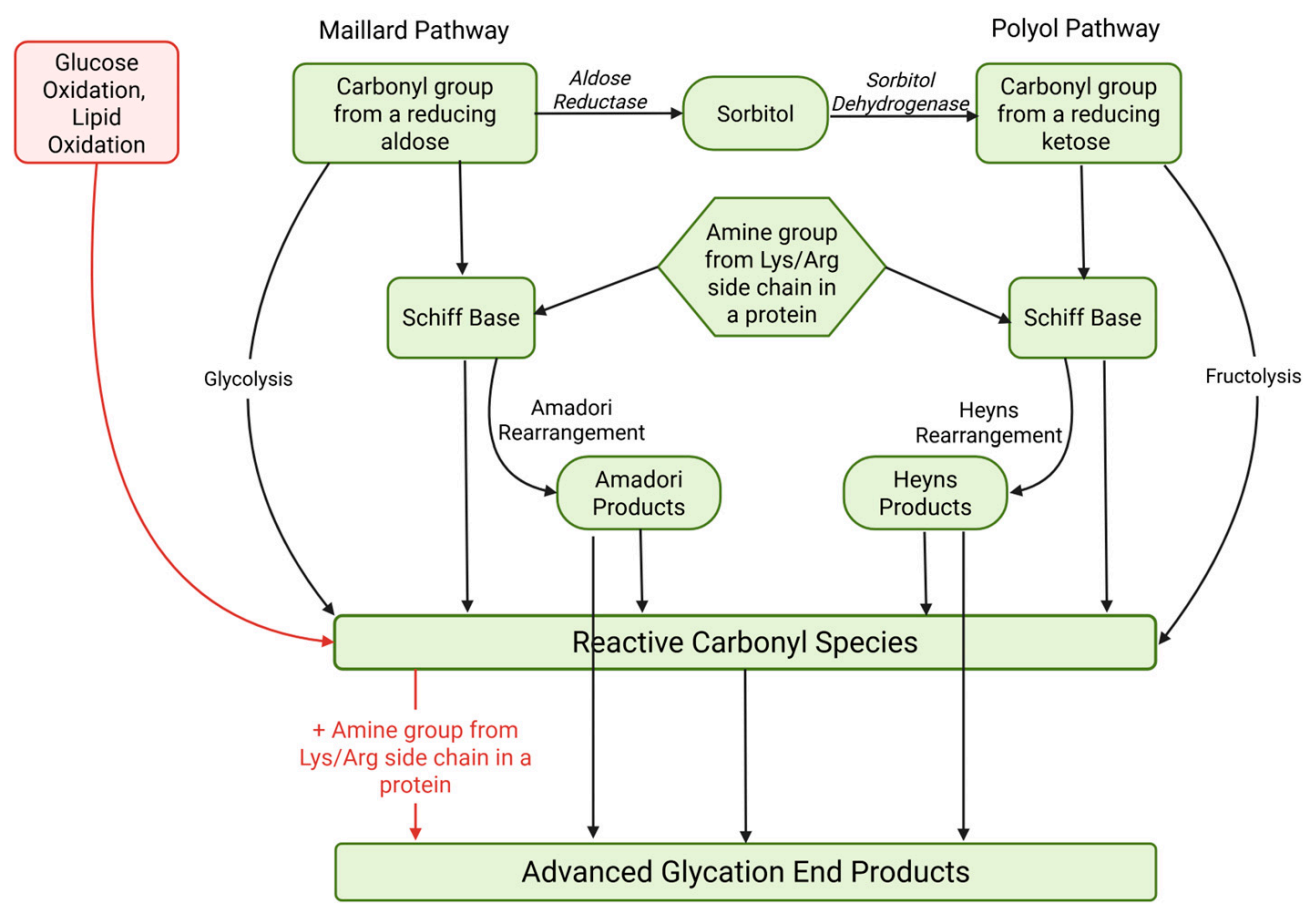
Overview of the Maillard reaction and the formation pathway of advanced glycation end products (AGEs). Adapted from Ref. [3]. Created in BioRender. Akalin Saygili, S. (2026) https://BioRender.com/2u5aifw (accessed on 6 August 2026).

The formation reactions of AGEs are summarized in Figure 1 [3]. AGE formation initiates with reducing aldose or ketose compounds in the cells. The carbonyl group source molecules participate in the formation of Schiff bases through either the Maillard or Polyol pathway. Following Amadori or Heyns rearrangements, the subsequent outcome involves the formation of reactive carbonyl compounds. The compounds created by Amadori rearrangement are considered as glucose-derived AGEs, while the Heyns products are considered as fructose-derived AGEs. These compounds are also called early glycation products [20]. Reactive carbonyl species undergo further processes, resulting in the formation of AGEs [3]. Reactive carbonyl species also generated through the oxidation of glucose and lipids can undergo subsequent reactions by combining with the amine group of lysine or arginine, ultimately leading to the formation of AGEs [21]. Advanced glycation end products exhibit various classifications based on their sources, precursor, chemical structure, fluorescence emission ability, and physiological importance [3].

The human body is exposed to AGEs through two main pathways: endogenous production and exogenous dietary intake. The formation of endogenous AGEs and their precursors occurs through the pathways depicted in Figure 1 [3]. The major stages of non-enzymatic glycation begin with the reaction between reducing sugars and free amino groups of proteins to form Schiff bases, followed by Amadori rearrangement. Subsequent oxidative and dehydration reactions generate reactive carbonyl species such as methylglyoxal (MGO), glyoxal (GO), and 3-deoxyglucosone (3-DG). These intermediates react with lysine and arginine residues to form stable AGEs, including Nε-carboxymethyl lysine (CML) and Nε-carboxyethyl lysine (CEL) [3]. In addition, the Polyol pathway and glycolysis play significant roles in the accumulation of endogenous AGEs [11].

Although both endogenous an exogenous AGEs ultimately contribute to the body’s AGE burden, they differ in their origins and determinants. Endogenous AGEs are primarily generated through hyperglycemia, oxidative stress and metabolic pathways, whereas exogenous AGEs originate predominantly from thermally processed foods. While endogenous production represents the major source of AGE accumulation, exposure to dietary AGEs remains modifiable through food selection and cooking practices.

Exogenous AGEs specifically mean the subset of AGEs that are obtained through dietary consumption or exposure to various environmental factors [11]. Dietary AGEs and their precursors are typically generated during food processing procedures to enhance the taste, quality, and shelf life of the food products [22]. In this process, the formation of AGEs and their precursors takes place through various non-enzymatic Maillard reaction pathways [23]. In the process of AGE formation, reducing sugars react with amino acids, leading to the formation of reversible Schiff base compounds called Amadori products. These Amadori products can rearrange via the Hodge pathway, producing AGEs through a series of reactions. Additionally, the Amadori product precursors can be broken down into reactive dicarbonyls through the Namiki pathway, which can also contribute to the formation of AGEs by reacting with amino acids [24,25]. Among the numerous types of AGEs mentioned, CML, CEL, and pentosidine are the most extensively studied [12,26].

As intracellular AGEs accumulate, they cross-link with cellular receptors and structural proteins, impairing their function and promoting oxidative stress and inflammation [27]. Elevated levels of oxidative stress, cytokines, acute phase proteins, as well as pro-inflammatory and inflammatory markers, pose an enlarged susceptibility to chronic diseases, including diabetes and cardiovascular diseases, among individuals. Through this mechanism, AGEs exert pathogenic effects within cells [28].

## 3. AGE Levels in Foods

AGE formation in foods is influenced by macronutrient composition, temperature, time, moisture, pH, and trace elements [7,29,30]. The analysis of AGE contents has predominantly focused on meat/meat products and grain products as food groups [2,15,31,32,33,34]. High-fat foods, particularly meats, tend to contain higher AGE levels, partly due to enhanced lipid peroxidation during cooking. The increased presence of protein and fat in such foods contributes to the overall higher AGE content in the diet [35]. Acidity and humidity are also an important factor in AGE formation. The pH level increases the reactivity and increases AGE formation [36]. The relatively low AGE content in vegetables and fruits can be attributed to the abundance of water in these foods, which aids in reducing AGE formation. Additionally, the presence of antioxidant vitamins and phytochemicals further contributes to the mitigation of AGE formation in these plant-based food items [37]. However, their AGE levels can increase during processes such as drying [38].

In the modern diet, food processing is recognized as an important source of dietary AGEs. However, the amount formed varies considerably depending on temperature, moisture, heating duration and food matrix characteristics. Cooking conditions substantially influence the formation of dietary AGEs, yet findings across the literature remain inconsistent and highly dependent on temperature, moisture, and heating duration [30]. Traditional dry-heat processes such as roasting, grilling, frying, and baking consistently yield higher AGE levels because they accelerate Maillard-driven glycoxidation under low-moisture, high-temperature conditions [30,37,39,40]. Moist-heat techniques, including boiling, steaming, and stewing, typically produce fewer AGEs, as water activity suppresses browning reactions and slows the progression from early glycation products to stable compounds such as CML and CEL [38,41,42]. Nevertheless, moisture alone does not fully explain the variability observed across studies. AGE formation is also affected by cooking duration, repeated heating and the food matrix composition. Furthermore, the relationship between processing conditions and glycation is often non-linear because polyphenols may exhibit concentration-dependent antioxidant or pro-oxidant behavior, and because interactions among food constituents can alter the availability of glycation.

Novel technologies have emerged as potential alternatives for reducing AGE formation, although current evidence remains limited and sometimes inconsistent. Sous-vide cooking generally restricts AGE accumulation through precise low-temperature processing, whereas microwave heating may reduce AGEs by shortening cooking time [38,43]. Air frying has also been reported to produce lower AGE levels than conventional deep frying, possibly due to reduced oil absorption and more efficient heat transfer [32]. Emerging approaches such as ohmic and infrared heating show promise for limiting AGE formation through rapid and uniform heating; however, further studies using standardized analytical methods are needed to confirm their effect [44]. While low-temperature and moisture-based methods appear favorable, the evidence base is constrained by methodological inconsistencies, differences in food composition, and variability in AGE quantification. More systematically designed studies are required to determine whether new cooking technologies offer a meaningful advantage over traditional methods in reducing dietary AGE exposure.

**Figure 2:**
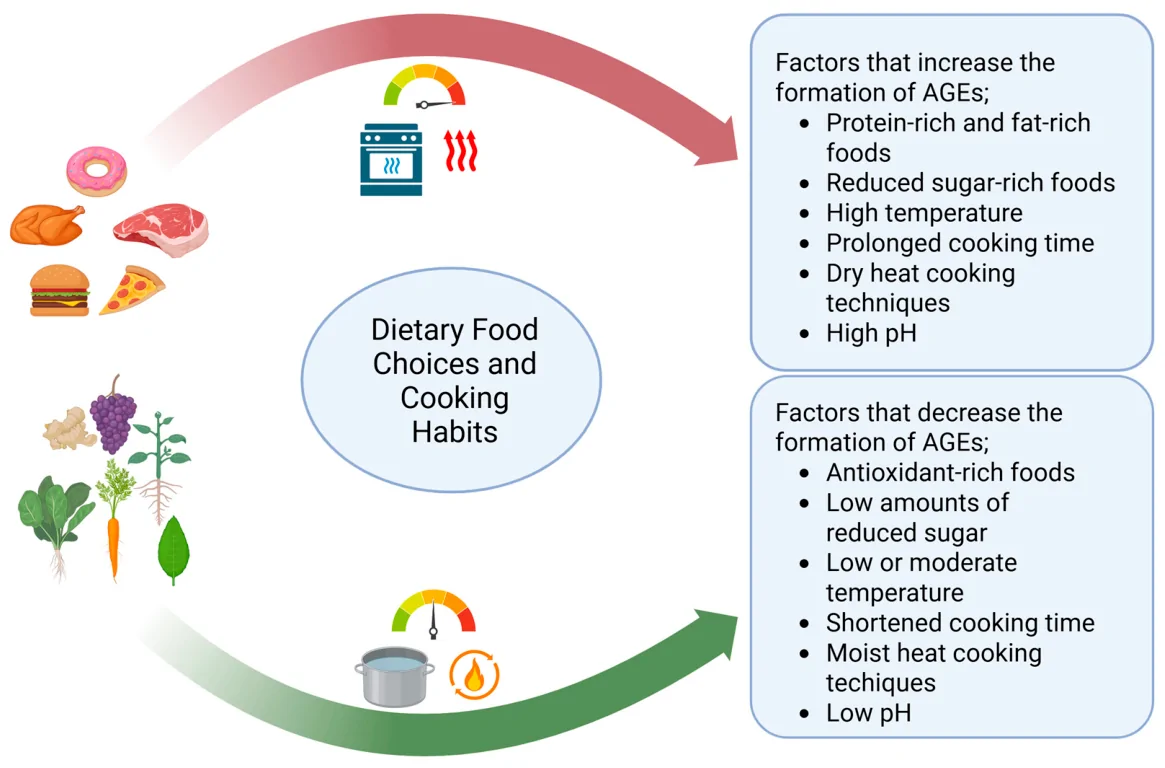
Factors influencing dietary intake of advanced glycation end products (AGEs) [6,37]. Created in BioRender. Akalin Saygili, S. (2026) https://BioRender.com/2gijyk3 (accessed on 6 August 2026).

Consequently, all components of the diet affect the intake of exogenous AGEs. Studies have shown that dietary AGE intake affects serum AGE levels [1,45]. Some dietary choices and cooking habits that influence AGE intake are summarized in Figure 2 [6,37].

Food-derived AGEs exhibit a wide range of compounds with varying chemical structures due to the diversity of precursors in foods. But only a few of them have been precisely identified and quantified in foods. CML, the most extensively studied AGE, is considered a reliable indicator of AGE accumulation in the body [29]. The amount of CML in some foods is summarized in Table 1. Table 1 clearly demonstrates that dry-heat cooking methods substantially increase CML concentrations. For example, grilled and pan-fried meat products consistently contain higher CML concentrations than boiled or stewed counterparts. Similar trends are observed in grain products and nuts, where thermal processing markedly increases AGE formation. These findings highlight the importance of cooking conditions as a determinants of dietary AGE exposure.

### Analytical Approaches for AGE Determination

Due to the considerable diversity of AGEs, their physical properties and polarities vary widely, making their purification prior to analysis challenging. Therefore, no universally accepted analytical method is currently available for all AGE species, and each method has its own advantages and limitations [22,46]. Moreover, no single AGE has been established as a gold-standard marker for assessing overall AGE levels. However, CML is among the most frequently used marker for determining AGE content in foods [38,47].

Several analytical methods have been used for AGE determination, including liquid chromatography–tandem mass spectrometry (LC-MS/MS), high-performance liquid chromatography (HPLC), gas chromatography–mass spectrometry (GC-MS), and immunoassay-based techniques such as enzyme-linked immunosorbent assay (ELISA) [35,47,48,49,50]. Among these, LC-MS/MS provides high sensitivity and specificity and is therefore widely used for the determination of AGEs in food matrices [47]. LC-MS/MS enables sensitive detection of AGEs and accurate quantification even at low concentrations, with low detection limits for CEL and CML [51]. The acquisition and maintenance of advanced LC systems are costly, which may limit their accessibility [52,53].

ELISA is also used to determine various AGE species [38,39]. However, the reliability of this method depends on the specificity and characterization of the target antibody, as cross-reactivity with other molecules may affect the results. ELISA methods have been evaluated for cross-reactivity against only a limited number of AGE-modified and unmodified structures [54,55]. For example, monoclonal antibody 6D12, considered selective for CML, was also found to cross-react with CEL [56]. Further research is therefore needed to improve the reliability of ELISA-based AGE determination. In addition, ELISA has been criticized for its relatively low accuracy and semi-quantitative nature [22,47,57]. ELISA is more cost-effective and may therefore be preferred in studies involving a large number of samples.

## 4. The Role of Dietary Polyphenols in Reducing AGE Levels

Before the growing interest in natural antiglycation strategies, several synthetic compounds, including aminoguanidine, pyridoxamine and metformin, were investigated for their ability to inhibit AGE formation through mechanisms such as carbonyl trapping, suppression of glycooxidation, and inhibition of protein cross-linking [22,58,59,60,61,62,63]. Despite their promising antiglycation activity, concerns regarding their safety, toxicity, and limited clinical efficacy have restricted their practical application [64,65,66,67,68]. Consequently, attention has shifted toward naturally derived compounds as safer and more feasible alternatives.

Polyphenols play a crucial role in preventing glycation due to their significant antioxidant capacity and capability to effectively scavenge free radicals [16,69]. Polyphenols are compound structures derived from natural sources and are commonly consumed as part of a healthy diet [70]. Natural antioxidants are primarily derived from plant-based sources, including fruits, vegetables, herbs, and spices, which are rich in phenolic compounds, vitamins, and carotenoids. Despite their natural origin, their application as food-grade antioxidants may be limited by factors such as sensory effects, appropriate dosage, and potential toxicological concerns [71].

In addition to exogenous dietary compounds, endogenous antioxidant defense systems play an important role in limiting AGE formation. Enzymatic antioxidants, including superoxide dismutase, catalase, and glutathione peroxidase, reduce oxidative stress and thereby suppress the generation of reactive carbonyl intermediates involved in glycation. Furthermore, the glyoxalase system, composed of glyoxalase I, glyoxalase II, and glutathione, detoxifies reactive dicarbonyl compounds such as methylglyoxal before they can react with proteins to form AGEs. Under conditions of persistent oxidative stress or impaired antioxidant defenses, these endogenous protective mechanisms become insufficient, leading to increased AGE accumulation. Dietary polyphenols may therefore complement endogenous antioxidant systems by reducing oxidative stress and scavenging reactive carbonyl species.

### 4.1. Mechanisms of Polyphenol-Mediated AGE Inhibition

Polyphenols inhibit AGE formation through multiple complementary biochemical pathways that span early, intermediate, and late stages of the Maillard reaction. These mechanisms collectively reduce the accumulation of reactive carbonyl species (RCS), suppress oxidative stress, and limit downstream AGE–Receptor for AGEs (RAGE) signaling [72]. One major mechanism involves carbonyl trapping, whereby polyphenols directly react with dicarbonyl intermediates such as MGO, GO, and 3-DG to form stable adducts and prevent their interaction with lysine and arginine residues [72]. Polyphenols also exert potent antioxidant effects by neutralizing reactive oxygen species (ROS), decreasing glycoxidation-derived radicals, and attenuating oxidative pathways that accelerate AGE formation [2,73]. In addition, many polyphenols chelate transition metals, including Fe^2+^ and Cu^2+^, thereby inhibiting metal-catalyzed oxidative steps involved in glycation [72,73]. Another key mechanism is the inhibition of protein glycation and cross-linking; polyphenols reduce early glycation reactions, such as Schiff base and Amadori product formation, and limit the generation of late-stage AGEs such as CML and CEL [74,75]. Finally, polyphenols may modulate AGE–RAGE signaling by downregulating RAGE expression or suppressing downstream inflammatory pathways including nuclear factor kappa B (NF-κB) activation, ultimately reducing AGE-mediated cellular injury [69,72,76].

By reviewing the existing literature, the most frequently investigated types of polyphenols and their main interaction mechanism have been summarized in Table 2. Numerous studies have been conducted to investigate the effects of polyphenols on AGEs. In addition to nutritional analyses, in vivo and in vitro studies have been extensively discussed in the literature to elucidate the mechanisms and outcomes associated with polyphenol intervention in AGE-related processes. Among polyphenol subclasses, flavonoids consistently exhibit the strongest antiglycation activity because they combine carbonyl trapping, antioxidant activity and modulation of AGE–RAGE signaling. In contrast, phenolic acids and stilbenes often exert more selective effects, suggesting that chemical structure is an important determinant of antiglycation efficacy.

### 4.2. Food Analysis Studies

Polyphenols influence AGE levels through two complementary pathways: by reducing AGE formation during food processing and by potentially suppressing endogenous AGE production after consumption [4,84].

Based on scientific investigations, the incorporation of polyphenol-rich foods or extracts into high-AGE-content foods has been shown to decrease the overall AGE content. The inhibitory effects shown in Table 2 indicate that different polyphenol subclasses reduce AGE formation depending on their structure and concentration [2,15,31,32,33,34,85,86]. A summary of related studies examining the impact of dietary polyphenols on AGE levels is presented in Table 3. According to Table 3, vegetable extracts and curcumin extract have been shown to exert significant effects on CML levels, particularly in meat products. The concentration of the phenolic compounds used also alters the inhibition of both CML and CEL to varying degrees. Polyphenols such as quercetin and chlorogenic acid are known to reduce AGE formation under in vitro conditions [74,87], and similar reductions in certain AGE markers have been reported in food applications, particularly in bakery products [31,33,34,86,88].

Although numerous studies have demonstrated that plant extracts and isolated polyphenols effectively inhibit AGE formation in various food systems, direct comparison among studies remains challenging because of substantial differences in extraction procedures, polyphenol composition, and application levels [2,15,31,32,33,34,85,86,88,89]. Extracts obtained using different solvents or processing conditions may vary considerably in both total polyphenol content and the relative abundance of individual bioactive compounds, making equivalent dose comparisons difficult. Moreover, many experimental studies express extract addition as a percentage of the food matrix or as purified compounds rather than standardized polyphenol concentrations, limiting comparisons with natural dietary exposure. Consequently, the concentrations required to achieve significant antiglycation effects in experimental food models may not always reflect those realistically obtained through routine consumption of polyphenol-rich foods.

The effectiveness of polyphenols also appears to be strongly influenced by the food matrix. In protein-rich systems such as meat and dairy products, inhibition is primarily associated with suppression of lipid oxidation and reactive carbonyl formation [2,15,49], whereas in carbohydrate-rich products including bread, cookies, and muffins, interactions with Maillard reaction intermediates become more prominent [31,33,34]. Differences in moisture content, pH, fat composition, heating intensity, and protein–carbohydrate interactions further modify polyphenol stability and their accessibility to reactive intermediates. Therefore, antiglycation efficacy observed in one food model cannot be directly extrapolated to other food systems.

From a practical perspective, these findings have important implications for food manufacturing. Foods subjected to intensive thermal processing, including processed meat products, bakery products, ready-to-eat cereals, and other ultra-processed foods, constitute major dietary sources of AGEs. Therefore, reducing AGE formation during food manufacturing represents an attractive preventive strategy to decrease dietary AGE exposure. In addition to optimizing processing conditions, incorporation of polyphenol-rich plant ingredients has emerged as a promising clean-label approach for limiting AGE formation during food production [37,38,88,90]. The studies conducted by Mildner-Szkudlarz et al. [34] and S. Zhang et al. [2] have demonstrated that the effects of extracts or polyphenols in inhibiting AGEs varies depending on the specific type used. Furthermore, the studies conducted by Lin et al., [33] Wang et al., [91] and Zhu et al. [32] have indicated that the inhibitory effects of polyphenols on AGE formation are influenced by the dosage employed. As demonstrated, the inhibitory impact of polyphenolic compounds on AGE content is concentration-dependent, though not following a linear pattern [34].

Polyphenols exert protective effects against lipid and protein oxidation and inhibit the formation of reactive dicarbonyl compounds, such as methylglyoxal and glyoxal, thereby suppressing AGE formation during food processing [2,15,31,32,33,85]. Their application has also been shown to reduce AGE formation during industrial processes such as pasteurization [92]. In the context of food processing, the incorporation of polyphenols not only reduces the intake of AGEs but also serves as a preventive measure against exogenous AGE production [32,33,91]. These findings suggest that the practical value of dietary polyphenols extends beyond demonstrating antiglycation activity under experimental conditions and increasingly supports their application as functional ingredients for reducing AGE formation during food processing. Future research should therefore focus on developing standardized polyphenol-enriched food formulations that simultaneously maximize AGE inhibition while maintaining nutritional quality, processing stability, and consumer acceptability. Such studies should combine standardized food processing models with comprehensive AGE profiling and sensory evaluation to facilitate industrial translation.

### 4.3. In Vitro and In Vivo Studies

In recent years, there has been a growing research focus on natural inhibitors of AGEs, with particular attention given to substances rich in polyphenols. Foods and extracts that possess a high content of polyphenols have been found to play a significant role in preventing glycation processes through several mechanisms. These include capturing glycation intermediates, inhibiting molecular aggregation, suppressing reactive oxygen species, scavenging reactive carbonyl intermediates, and competitively binding with the free amino acid residues of proteins [18,69,93]. Studies investigating Chilean bean leaf extracts, Camellia oleifera peels, peanut skin polyphenols, and black chokeberry have consistently reported the positive impact of these polyphenols on AGE levels [16,18,69,94]. Simultaneously, these polyphenols exhibit anti-inflammatory activity by reducing the levels of pro-inflammatory cytokines. Moreover, they demonstrate a protective effect against inflammation induced by nitric oxide or AGEs in various cellular models [18,69].

It is known that the efficacy levels may vary among different species within the same food group based on their phenolic content. Moreover, certain polyphenols present within the composition of a specific food group have been suggested to exhibit greater efficacy in AGE inhibition compared to others [94]. For instance, a study comparing the effects of extracts from four distinct tomato species on AGE inhibition revealed variations in their inhibitory potential. Although extracts from tomatoes generally share similar chemical structures, their phenolic contents may differ. The study findings indicate that extracts derived from Black Prince tomatoes display a more definite inhibition of AGE formation compared to the other tomato species [87]. In a study conducted by Qin et al. [19], it was observed that oat polyphenols, specifically luteolin and apigenin, exhibited inhibitory effects on AGE formation. Another study revealed that a plant extract containing significant amounts of chlorogenic acid, quercetin, and rutin exhibited a remarkable ability to capture MGO, a key reactive carbonyl implicated in the formation of AGEs. This increased MGO capture capacity effectively impedes AGE formation [95]. Jin et al. [17] conducted a study to explore the impact of tea polyphenols on fish meat during processing. The findings of the study revealed that tea polyphenols effectively mitigate AGE formation by binding with free lysine and arginine residues [17].

In vitro studies offer controlled conditions to clarify specific antiglycation mechanisms, yet they oversimplify biological reality; they ignore digestion, phase II metabolism, microbial transformation, and the low physiological concentrations at which polyphenols actually circulate. Consequently, in vitro outcomes often overestimate true biological efficacy. In vivo models, by contrast, incorporate absorption, metabolism, and systemic distribution, providing more realistic insights, but introduce variability due to species differences, heterogeneous metabolic responses, and challenges in controlling dietary and environmental factors. Thus, in vitro studies are mechanistically precise but biologically limited, while in vivo studies are physiologically relevant but experimentally constrained.

## 5. Bioavailability of Polyphenols and Its Clinical Outcomes

Despite their potent antiglycation activity in vitro, the clinical relevance of polyphenols is strongly influenced by their limited bioavailability and extensive metabolic transformation. Most dietary polyphenols undergo rapid phase II conjugation (glucuronidation, sulfation, and methylation) in the small intestine and liver, resulting in reduced concentrations of free aglycone forms in circulation [96]. Importantly, many in vitro antiglycation studies evaluate polyphenols at concentrations ranging from tens or hundreds of micromolar to even millimolar levels, whereas the circulating concentrations of unconjugated aglycones following dietary intake are generally in the nanomolar range and only rarely approach low micromolar levels after high-dose supplementation. This substantial discrepancy raises concerns regarding the physiological relevance of many in vitro findings, as the concentrations required to inhibit AGE formation experimentally are unlikely to be achieved under normal physiological conditions [97]. In addition, colonic microbiota metabolize polyphenols into smaller phenolic acids, urolithins, and other low-molecular-weight metabolites, which may exhibit biological activities distinct from those of their parent compounds [98]. Moreover, because circulating polyphenols predominantly exist as glucuronidated, sulfated, or methylated metabolites rather than as parent aglycones, it remains uncertain whether these metabolites possess comparable antiglycation activity [99]. Consequently, the biological effects observed in vivo are more likely to be mediated by conjugated and microbial-derived metabolites than by the native polyphenol aglycones commonly used in cell-free and cell culture experiments. However, the antiglycation activities of many of these circulating metabolites remain insufficiently characterized, representing an important knowledge gap that limits the clinical translation of current experimental evidence. These metabolic processes, together with rapid renal clearance, markedly reduce systemic exposure to intact polyphenols and consequently limit their in vivo antiglycation efficacy [80].

Furthermore, the polyphenol composition of foods varies considerably depending on the extraction method used, leading to differences in the type and concentration of bioactive compounds and consequently in their biological effects. Advances in next-generation extraction technologies aimed at enhancing the stability, concentration, and bioavailability of dietary polyphenols may therefore help strengthen the clinical evidence supporting their antiglycation potential [100].

Human evidence on polyphenol-mediated reductions in AGE accumulation remains limited but provides some preliminary support. Several small-scale clinical trials report improvements following polyphenol intake. Flavonoids and ellagitannins appear to exert moderate reductions in circulating MGO levels, as demonstrated in a study where four weeks of citrus and pomegranate polyphenol supplementation in healthy older adults resulted in a 9.8% decrease in plasma MGO concentrations [101]. However, not all findings are consistent; a six-week intervention combining polyphenols with L-citrulline in prehypertensive individuals did not produce significant changes in skin AGE levels [102]. Quercetin supplementation has shown small but meaningful reductions in plasma CML and improvements in oxidative stress biomarkers in adults with metabolic risk factors [103,104]. In another four-week clinical trial, quercetin lowered plasma MGO by 11% from baseline, whereas GO, 3-DG, and both free and protein-bound AGEs remained unchanged; notably, epicatechin had no measurable impact on any dicarbonyls or AGE markers [105]. Resveratrol trials report reductions in inflammation and oxidative stress, although findings regarding AGE markers remain inconsistent [106].

Taken together, current clinical evidence suggests that the promising antiglycation effects consistently observed in vitro have not yet been fully translated into clinical efficacy [99]. This gap is likely attributable to the low systemic availability of intact polyphenols, extensive metabolic transformation, interindividual differences in gut microbiota composition, and the use of experimental concentrations that are difficult to achieve under physiological conditions [107]. Future clinical trials should integrate pharmacokinetic data and evaluate both circulating conjugated metabolites and microbiota-derived metabolites at physiologically relevant concentrations.

## 6. Effects of AGE Levels on Health

**Figure 3:**
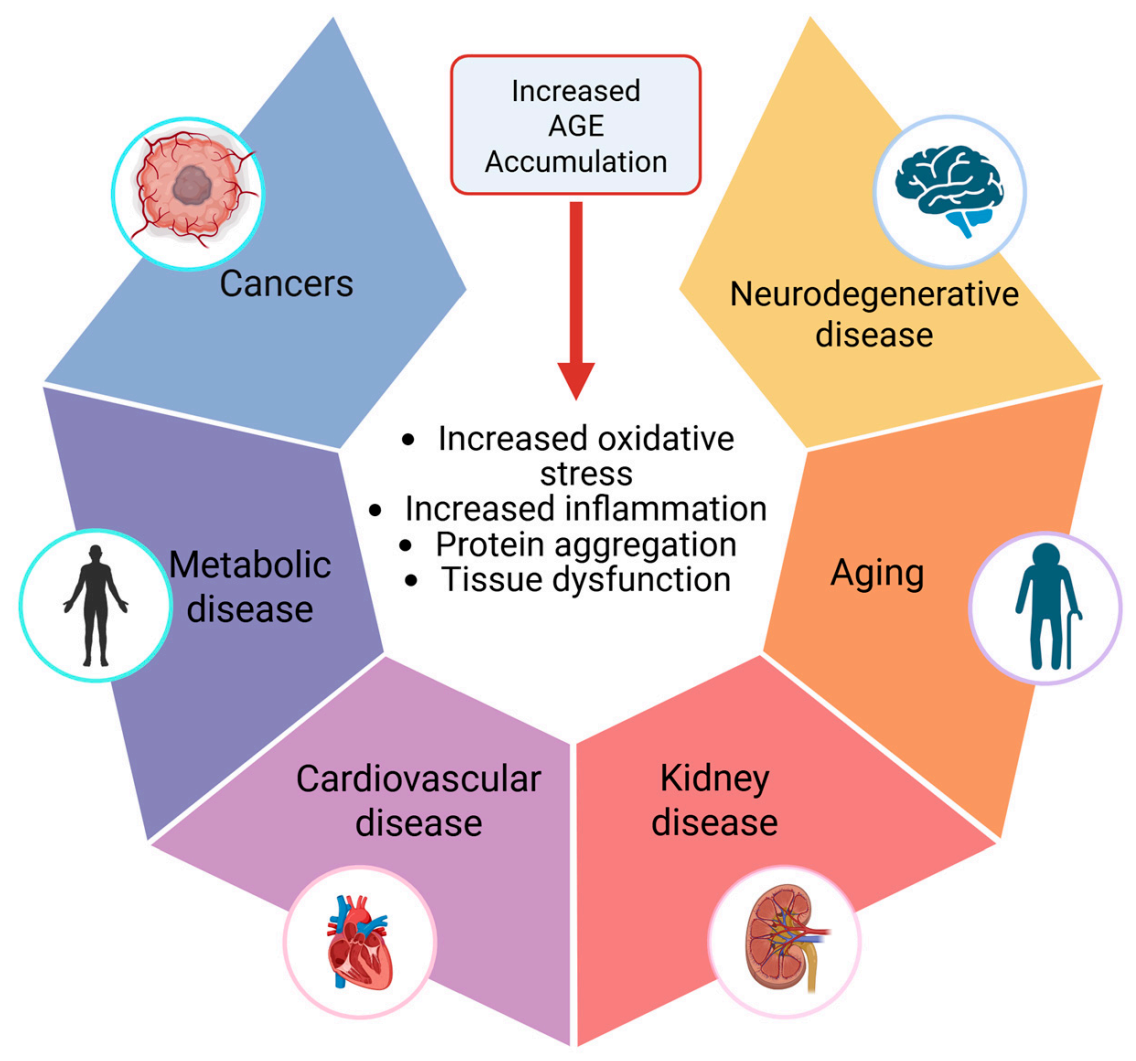
Effects of AGE accumulation on health. Created in BioRender. Akalin Saygili, S. (2026) https://BioRender.com/ee5aze3 (accessed on 6 August 2026).

Humans are continuously exposed to both endogenously produced and diet-derived AGEs. The accumulation of AGEs in the body can lead to important health problems in the long term. This process is amplified by oxidative stress, free radicals, impaired detoxification of reactive carbonyl species, and reduced renal clearance. Increasing AGE levels in the body promotes alterations in protein function, stimulates macrophages and increases the formation of inflammatory factors such as prostaglandin E2, nitric oxide, and interlokin-6. It is known that AGEs and dicarbonyls play a role in the pathogenesis of many diseases such as those associated with the aging process, insulin resistance, diabetes, metabolic syndrome, cardiovascular diseases, atherosclerosis, chronic kidney diseases, neuropathy and Alzheimer’s [60,108,109]. Diseases commonly associated with AGE burden in the body and their possible causes are summarized in Figure 3.

### 6.1. Diabetes Mellitus

Elevated AGE levels impair insulin-mediated glucose uptake and contribute to dysregulated glucose metabolism. At the same time, increased AGE–RAGE interaction also prevents glucose uptake by increasing intracellular reactive oxygen species [110]. Reducing oxidative stress and non-enzymatic glycation has positive effects on the pathology of diabetes [9]. Thus, high AGE levels may play a role in the development of diabetes-related complications. In relation to this matter, studies have shown that plant extracts with high polyphenol content have an inhibitory effect on glycation of alpha-glucosidase, aldose reductase, and bovine serum albumin. And it has been determined that they play an important role in the inhibition of reactive carbonyl species [31,111,112]. Therefore, plants/extracts with polyphenol content are thought to have a role in the prevention of hyperglycemia and related complications.

Human studies support these mechanistic findings. Higher circulating CML and CEL levels have been associated with increased risk of type 2 diabetes, poorer glycemic control, and elevated glycated hemoglobin (HbA1c) [113]. In addition, several intervention trials in individuals with diabetes have reported little or no improvement in glycemic or lipemic parameters following a low-AGE diet [13]. Dietary AGE intake also correlates with markers of metabolic dysfunction in individuals with metabolic risk factors [114]. However, these data are largely observational, and controlled intervention studies remain limited, preventing firm conclusions regarding causality.

### 6.2. Cardiovascular Diseases

The accumulation of AGEs in the body is closely related to cardiovascular diseases. AGEs cross-link extracellular matrix proteins and cause vascular atherosclerosis. Additionally, AGEs affect the functions of vascular smooth muscle cells. AGE–RAGE signaling increases the production of pro-inflammatory cytokines and vascular adhesion molecules, disrupting endothelial homeostasis [10]. The utilization of phenolic compounds with foods has positive effects on cardiovascular disease as well as reducing AGE production. In a study, epigallocatechin gallate exhibited anti-cardiac hypertrophy, anti-myocardial infarction, and an anti-atherosclerosis effect by reducing low-density lipoprotein (LDL) cholesterol, and inhibiting nuclear factor kappa b and myeloperoxidase activity [115]. Similarly, resveratrol from the stilbenes group is known to have a role in the prevention of cardiovascular disease and atherosclerosis [4].

Human data also support these associations. Higher circulating AGE levels and increased tissue AGE accumulation have been linked with arterial stiffness, coronary artery calcification, and greater cardiovascular mortality in observational cohorts [113,114]. However, these relationships are largely associative, and intervention studies reducing dietary AGE intake have yielded inconsistent effects on vascular markers, suggesting that the clinical impact of AGE reduction may be influenced by baseline cardiovascular risk, intervention duration, and individual metabolic responses.

### 6.3. Kidney Diseases

AGEs are closely associated with the development and progression of kidney diseases, largely due to impaired renal clearance and increased local formation of reactive carbonyl compounds. Certain types of AGEs, particularly methylglyoxal-derived dihydroimidazolone, carboxymethyllysine, and glucosepane, contribute to the development of chronic kidney disease [8]. In individuals with chronic kidney disease, AGE accumulation may increase due to impaired removal of AGEs from the body. A positive correlation was found between AGE level and mortality risk as a result of non-invasive measurement of skin AGE levels in individuals with chronic kidney disease [116]. Excessive AGE accumulation contributes to diabetic nephropathy by increasing dicarbonyl stress. While AGE accumulation triggers kidney diseases, decreased renal clearance also increases AGE accumulation. Accordingly, kidney diseases can promote the accumulation of AGEs, and conversely, the accumulation of AGEs can contribute to the development or progression of kidney diseases as well as other associated secondary diseases [8]. AGEs contribute to key pathogenic mechanisms in chronic kidney disease, but the clinical benefits of reducing AGE exposure remain uncertain [117]. Well-controlled long-term trials are needed to determine whether dietary or therapeutic AGE-lowering strategies can meaningfully slow renal decline or improve clinical outcomes in kidney disease.

Higher circulating AGEs and CML concentrations have been associated with faster chronic kidney disease (CKD) progression, increased albuminuria, and greater risk of renal complications in observational studies [118,119]. However, clinical data remain mostly associative, and interventions targeting dietary AGE reduction or AGE–RAGE signaling pathways have produced inconsistent results. These findings suggest that the impact of AGEs on kidney disease may vary depending on baseline renal function, and the degree of existing renal injury.

### 6.4. Neurological Diseases

AGEs are implicated in the progression of several neurological disorders through mechanisms involving oxidative stress, mitochondrial dysfunction, and chronic inflammation. Alzheimer’s disease is characterized by progressive cognitive decline and neurodegeneration. High AGE formation affects the development of Alzheimer’s by affecting oxidative stress and the neuroimmune system. In a study, AGE molecules were detected in senile plaques in neurofibrillary tissues and hippocampal tissues [120]. Recent evidence highlights a strong link between Alzheimer’s disease and metabolic dysfunction, particularly diabetes. Cognitive disorders are also observed in individuals with insulin resistance and impaired glucose control. It is determined that the concentration of AGEs in body tissues and serum is high with diabetes [121]. In another longitudinal study, it was determined that serum AGE levels would be effective in cognitive decline in the long term [122]. Therefore, increased serum AGE markers and AGE exposure has negative neurological effects [123].

High AGE levels may directly or indirectly cause neurological diseases but current evidence is predominantly experimental and observational. More mechanistic human studies and controlled clinical trials are needed to determine whether lowering AGE burden can meaningfully affect neurological outcomes.

### 6.5. Aging

With the progression of aging, there is a natural process characterized by an increase in cellular oxidative stress and a decline in the regenerative capabilities of tissues and cells throughout the body. In addition, high AGE levels cause cross-linking and aggregation in proteins, impairing cell signaling and causing cellular damage. This phenomenon holds significant implications in the pathophysiology of aging [124].

Choi et al. demonstrate that both natural and synthetic agents with antiglycation or antioxidant activity play a role in improving skin aging parameters [125]. Emerging data also link AGEs to declines in muscle health. In experimental models, methylglyoxal accelerated muscle atrophy through activation of catabolic pathways and reduced myogenic signaling, effects that were prevented by aerobic exercise. In humans, higher skin autofluorescence, reflecting greater AGE burden, was associated with increased insulin resistance, elevated CML levels, and poorer muscle strength and physical performance [126]. As individuals age, there is a decline in muscle strength, muscle mass and overall body function. However, if this decrease is faster than the normal aging process, it is called sarcopenia. According to Granic et al. [127], the amount of AGEs in circulation is positively associated with the risk of sarcopenia. In light of these findings, AGE accumulation may contribute not only to structural tissue aging but also to declines in physical capacity, although causal relationships in humans remain unclear.

### 6.6. Cancers

AGEs may contribute to carcinogenesis through persistent activation of oxidative and inflammatory pathways. AGE–RAGE signaling promotes ROS generation, NF-κB activation, and proliferation-associated gene expression, creating a microenvironment that can support tumor initiation and progression. As reported in a study, polyphenol-rich extracts from brown algae, particularly phlorotannins, not only inhibited AGE formation in both in vivo and in vitro models but also induced autophagy and pro-apoptotic pathways in cervical cancer cells. These findings suggest that some polyphenols may influence cellular pathways associated with both glycation and tumor development. However, the potential health benefits of reducing AGE exposure are more likely mediated through attenuation of AGE–RAGE signaling, oxidative stress and chronic inflammation than through direct anticancer effects. Accordingly, lowering dietary AGE intake should be considered a supportive preventive strategy rather than a direct therapeutic approach against cancer [128].

Experimental studies show that AGEs enhance DNA damage, impair apoptotic responses, and facilitate metastatic behavior in various cancer models. In a study, some RAGE gene variants were found to be associated with the risk of cancer [129]. Similarly, plasma CML levels are higher in individuals with cancer [130]. Human data indicate higher circulating AGEs or increased tissue AGE accumulation in several cancer types, but these findings are largely associative and may reflect underlying metabolic dysfunction rather than a direct causal role [5]. Evidence from clinical or dietary intervention studies is inconsistent [131,132]. AGEs appear to influence pathways relevant to tumor biology, yet their contribution to cancer development in humans remains uncertain and requires more mechanistic and longitudinal research.

Although observational studies have consistently reported positive associations between dietary AGE intake and chronic diseases such as diabetes, cardiovascular disease, and certain cancers, these findings should be interpreted with caution. Diets rich in AGEs are often characterized by a higher consumption of red and processed meats, saturated fats, refined carbohydrates, and ultra-processed foods, all of which are established risk factors for chronic diseases independent of AGE exposure. Consequently, it remains difficult to distinguish the specific contribution of dietary AGEs from the overall dietary pattern and lifestyle characteristics of the study populations. Furthermore, residual confounding related to obesity, physical activity, smoking, socioeconomic status, and total energy intake cannot be completely excluded in observational studies, limiting causal inference. Therefore, although epidemiological evidence supports an association between dietary AGEs and chronic disease risk, current data are insufficient to establish a direct causal relationship. Well-controlled intervention studies are required to clarify the independent contribution of dietary AGEs to disease development.

## 7. Conclusions

Reducing both endogenous and dietary exposure to advanced glycation end products is essential for mitigating glycation-related metabolic stress and long-term health risks. A diet rich in plant-based polyphenols combined with appropriate cooking practices can help lower dietary AGE intake. Among polyphenol subclasses, flavonoids generally show the most consistent antiglycation activity, while phenolic acids, stilbenes, and lignans provide variable but promising effects depending on structure, dose, and food matrix. Culinary strategies such as lower-temperature cooking, moist-heat methods, and emerging approaches including sous-vide and air frying represent practical tools for reducing AGE formation in foods.

In contrast, the primary bottlenecks hindering the in vivo efficacy of polyphenols remain their poor systemic bioavailability—driven by extensive intestinal and hepatic phase II conjugation processes (glucuronidation, sulfation, and methylation)—and the diverse metabolic transformations carried out by the colonic microbiota. Furthermore, the inability of in vitro models to fully replicate complex biological environments, combined with the profound heterogeneity of AGE quantification methodologies (e.g., ELISA versus advanced chromatographic methods) across clinical trials, complicates the establishment of definitive causal relationships. Therefore, the therapeutic relevance of AGE-lowering dietary strategies has not yet been conclusively demonstrated in long-term human trials.

Future research should move beyond descriptive evaluations and adopt standardized experimental designs that improve comparability across studies. This includes reporting both total and individual polyphenol concentrations, using physiologically relevant doses, simultaneously quantifying multiple AGE biomarkers (e.g., CML, CEL, MG-H1, and dicarbonyl compounds) with LC-MS/MS, and evaluating identical polyphenols across different food matrices under standardized processing conditions. In addition, integrating food processing experiments with in vitro digestion models and pharmacokinetic assessments in human intervention studies would improve the translation of experimental findings into practical dietary recommendations. Strengthening these areas will support the development of more targeted dietary recommendations and help determine whether reducing AGE exposure can meaningfully influence chronic disease risk and overall health.

## Figures and Tables

**Table 1:** Nε-carboxymethyl lysine (CML) levels of some selected food items [38].

Food Item	CML Content (kU/100 g) *	Food Item	CML Content (kU/100 g) *
**Meat and Meat Products**		**Grains/Grain Products/Legumes**	
***Beef***		Bagel	107
Beef, ground, boiled	1538	Bagel, toasted	167
Beef, ground, pan browned	3833	Biscuit, refrigerator, uncooked	823
Beef, ground, 20% fat, pan browned	4928	Biscuit, refrigerator, oven-baked	1343
Beef, roast	6071	Bread, white, slice	83
Beef, steak, grilled 4 min	7416	Bread, white, slice, toasted	107
Beef, steak, pan fried	10,058	Bread, whole wheat, slice	103
Beef, stewed	2657	Bread, whole wheat, slice, toasted	137
***Poultry***		Beans, red kidney, raw	116
Chicken, boiled	1123	Beans, red kidney, cooked	298
Chicken, roasted	6020	Rice, white, quick cooking	9
Chicken, fried	7390	Rice, white, pan toasted	32
Chicken, breast, boiled	1210	***Snacks***	
Chicken, breast, grilled	4849	Chips, corn	503
Chicken, breast, pan fried	4938	Chips, potato	450
Chicken, breast, poached	1101	Bar, granola, chocolate chunk	507
Chicken, breast, roasted	6639	Bar, granola, peanut butter and choc chunk	3177
Turkey, breast, roasted	4669	Cookie, meringue, homemade	797
Turkey, breast, smoked, seared	6013	Dark chocolate	1777
***Pork***		Milk chocolate	1500
Pork, chop, raw	1188	**Fatty foods**	
Pork, chop, pan fried	4752	***Fats and Oils***	
Pork, roast	3544	Butter, whipped	26,480
***Fish/Seafood***		Margarine	17,520
Salmon, raw	528	Oil, canola	9020
Salmon, microwaved	954	Oil, corn	2400
Salmon, poached	1801	Oil, olive	11,900
Salmon, steamed	1212	Oil, peanut	11,440
Salmon, smoked	572	Oil, sunflower	3940
Salmon, fillet, boiled	1082	***Nuts***	
Salmon, fillet, broiled	3347	Almonds, blanched slivered	5473
Salmon, pan fried	3083	Almonds, roasted	6650
Shrimp, marinated raw	1003	Cashews, raw	6730
Shrimp, marinated, grilled	2089	Cashews, roasted	9807
**Milk and Milk Products**		Chestnut, raw	2723
Milk, whole (4% fat)	5	Chestnut, roasted	5353
Cheese, American	8677	Peanuts, dry roasted	6447
Cheese, cheddar	5523	Peanuts, roasted in shell	3440
Cheese, feta	8423	Sunflower seeds, raw, hulled	2510
Cheese, mozzarella	1677	Sunflower seeds, roasted	4693
Cheese, parmesan	16,900		
Cream cheese	8720		

* Values are based on the CML ELISA database reported by Uribarri et al. [38]. While chromatographic methods provide greater analytical precision, ELISA-based units remain useful for comparing AGEs across diverse food products and dietary datasets. Bold words represent food groups that share similar characteristics.

**Table 2:** Overview of the most frequently studied polyphenol subclasses and their primary antiglycation mechanisms.

Polyphenol Subclasses	Compounds	Main Antiglycation Mechanism	Relative Potency	References
Phenolic acids	Ferulic acids, coumaric acids, chlorogenic acids, caffeic acid, vanillic acids, gallic acid	Antioxidant: ROS scavenging, inhibition of lipid oxidation. Carbonyl-related: Reactive carbonyl trapping, protection of protein amino groups.	Moderate to strong	[64,77,78]
Flavonoids	Catechins, luteolin, rutin, quercetin, epigallocatechin gallate, daidzein, naringenin, myricetin	Antioxidant: ROS scavenging. Carbonyl-related: MGO/GO trapping. Protein glycation: Inhibition of glycation cascade. ROS scavenging	Strong	[79,80,81]
Stilbenes	Resveratrol, 2,3,5,4′-tetrahydroxystilbene-2-O-β-D-glucoside (THSG), oxyresveratrol, piceatannol	Antioxidant: ROS inhibition. Carbonyl-related: Reactive dicarbonyl trapping. Cell signaling: Anti-inflammatory activity, RAGE suppression.	Moderate	[64,82,83]
Lignans	Secoisolariciresinol, sesame-derived lignans	Antioxidant: Free radical scavenging, antioxidant-based antiglycation. Carbonyl-related: Inhibition of dicarbonyl precursor formation. Cell protection: Reduction of AGE-induced cellular toxicity.	Limited to moderate	[64,73]

**Table 3:** Studies investigating the effects of polyphenolic content addition on AGE formation in foods.

Food Type	Added Polyphenol/Extract	Processing Condition *	Key Findings	References
Roasted *Eleutheronema tetradactylum*	Tea extracts	1. Control group: Fish samples cut to the dimensions of 4 cm × 5 cm × 0.5 cm 2. Tea group: Fish samples marinated with 1% (*w*/*w*) tea extract for 45 min All samples roasted in oven and heated at 240 °C for 10 min on each side.	Tea extract reduced CML and CEL by 24% and 35% respectively.	[66]
Roasted mackerel	Vegetable extracts (celery, carrot, and yam)	1. Control group (50 g minced fish + 2 g soybean oil) 2. Yam group: Control group+1% *w*/*w* yam extract 3. Celery group: Control group+1% *w*/*w* celery extract 4. Carrot group: Control group+1% *w*/*w* carrot extract All samples were roasted in preheated oven at 240 °C for 20 min on each side.	All three vegetable extract treatments had a significant effect on CML inhibition. However, only yam extract exerted a significant inhibitory effect on CEL concentrations.	[2]
Roasted beef patties	Ginger and curcumin	1. Control group: 40 g ground beef 2. Ginger with different addition levels (0.5%, 1.0%, and 1.5%) was added to ground beef. 3. Curcumin with different addition levels (0.005%, 0.010%, and 0.015%) was added to ground beef. All samples were roasted at 225 °C for 10 min on each side.	The levels of free CEL, bound CML, and bound CEL gradually decreased in all samples with the addition of ginger and curcumin.	[13]
Cookies	Rooibos extract	1. Control group: Cookies with water, sugar, salt, white flour, baking powder, and canola oil 2. Test groups: 0.5 or 1% (*w*/*w*) of the dried extract of rooibos were mixed into the cookie dough. All samples were baked at 200 °C for 16 min in an oven.	In samples with rooibos extract, multiple AGE markers were inhibited. The inhibitory effect exhibited an ascending trend as the ratio of added rooibos increased.	[31]
Chicken breast	Grape seeds extract	1. Control group (chicken breast with sugar solution) 2. Test groups: Grape seeds extract was added to sugar solution with 0.0, 0.2, 0.5, 0.8, and 1.0 g/kg, respectively. Prepared solutions smeared on the surface of chicken breast and cooked in air fryer or deep fried in peanut oil at 180 °C for 3 min. Control group was not cooked.	Inhibition of CML and CEL levels was observed in all extract-added samples. The optimal method for CML and CEL inhibition was found to be the addition of 0.5 g/kg grape seed extract.	[32]
Baked milk and Baked yogurt	Resveratrol	1. Control group (no addition) 2. Test groups: Baked milk and baked yogurt with different levels of resveratrol addition (0,01 µM, 0,1 µM, 1 µM, 10 µM, 100 µM) Baked milk samples were obtained by boiling the milk at 98 °C for 210 min. Baked yogurt samples obtained by fermentation of this milk.	The addition of resveratrol at varying levels changed the contents of CML and CEL. The addition of 100 µM resveratrol did not significantly affect CML and CEL contents.	[62]
Steamed bread	Quercetin	1. Control group (with soft flour, fine salt, sugar, dry yeast and water) 2. Test groups: Added different levels (0.05, 0.10, 0.20%) of quercetin into soft flour. All dough samples (55 g) were placed in a proofer for 45 min and 10 min steaming in a steamer.	It was determined that the levels of AGE inhibition gradually increased in all samples with the addition of quercetin.	[33]
Bread	Catechin, Quercetin, Gallic acid, Ferulic acid, Caffeic acid	1. Control group (with wheat flour, water, fresh yeast, fine salt) 2. Test groups: Five different phenolic compounds were added at the level of 0.1, 0.5, 1.0, and 2.0 g per 100 g flour. All dough samples were placed in an incubator for fermentation and baked at 210 °C for 20 min.	The phenolic compounds showed the strongest inhibitory effect at the lowest concentration (0.1 g/100 g of flour), except for ferulic acid.	[34]
Muffins	Grape powder	1. Control group (with wheat flour, water, sugar, fat, nonfat dry milk powder, baking powder, dry egg white, salt) 2. Test group: Grape powder replacing 20% of the wheat flour in the muffin recipe. All dough samples (60 g) baked in a preheated oven at 180 °C for 20 min.	The addition of 20% grape powder to the products has been determined to be sensory acceptable and significantly decreases CML levels.	[63]
Cookies	Naringenin Quercetin Epicatechin Chlorogenic acid Rosmarinic acid	1. Control group (with double-deionized water, salt, baking powder, sugar, canola oil, and white flour). 2. Test group: All ingredients were mixed with 0.25% (*w*/*w*) of phenolic compounds (naringenin, quercetin, epicatechin, chlorogenic acid, and rosmarinic acid) All samples baked in an oven at 200 °C for 10 min.	Quercetin was most effective against fluorescent AGE formation, followed by naringenin, rosmarinic acid, and epicatechin. No significant effect of chlorogenic acid on AGE inhibition was found.	[65]

* Units reported as in the original publications.

## Data Availability

No new data were created or analyzed in this study. Data sharing is not applicable to this article.

## Abbreviations

The following abbreviations are used in this manuscript:

AGEs	Advanced glycation end products
MGO	Methylglyoxal
GO	Glyoxal
3-DG	3-deoxyglucosone
CML	Nε-carboxymethyl lysine
CEL	Nε-carboxyethyl lysine
RCS	Reactive carbonyl species
ROS	Reactive oxygen species
RAGE	Receptor for AGEs
NF-κB	Nuclear factor kappa B
THSG	2,3,5,4′-tetrahydroxystilbene-2-O-β-D-glucoside
HbA1c	Glycated hemoglobin
LDL	Low-density lipoprotein
CKD	Chronic kidney disease
°C	Degrees Celsius
